# Focus on blood pressure levels and variability in the early phase of acute ischemic stroke with hypertension and carotid stenosis

**DOI:** 10.1111/jch.14385

**Published:** 2021-11-16

**Authors:** Mingli He, Bing Cui, Jin'e Wang, Xiao Xiao, Taotao Wu, Mingyu Wang, Ru Yang, Bo Zhang, Bingchao Xu, Xiaobing He, Guanghui Zhang, Xiaoqin Niu, Zaipo Li, Bei Wang, Bei Xu, Rutai Hui, Yibo Wang

**Affiliations:** ^1^ The Affiliated Lianyungang Hospital of Xuzhou Medical University Jiangsu China; ^2^ State Key Laboratory of Cardiovascular Disease, Fuwai Hospital National Center for Cardiovascular Diseases Chinese Academy of Medical Sciences and Peking Union Medical College Beijing China; ^3^ College of Medical Science China Three Gorges University Yichang Hubei China; ^4^ The Neuroelectrophysiology Department Lianyungang Hospital Lianyungang Jiangsu China; ^5^ The Medical Imaging Department Lianyungang Hospital Lianyungang Jiangsu China

**Keywords:** blood pressure, blood pressure variability, carotid artery stenosis, cerebral blood flow

## Abstract

To investigate the optimal blood pressure (BP) levels and relative importance of BP and BP variability in the early phase of acute ischemic stroke (AIS) for hypertensive patients with carotid artery stenosis (CAS). A single‐center cohort study included 750 AIS patients with hypertension and tests were performed for CAS. Participants were categorized to Group 1 (SBP < 140 mm Hg and DBP < 90 mm Hg), Group 2: (SBP: 140–159 mm Hg and or DBP: 90–99 mm Hg), and Group 3: (SBP ≥160 mm Hg and/or DBP ≥100 mm Hg) according to the guidelines. The associations of mean BP levels and variability with outcomes (recurrent stroke, all‐cause death and the composite cardiovascular events) at 6 months were analyzed by Cox proportional hazard models. The associations of BP variability with BP levels and cerebral blood flow (CBF) were analyzed by linear regression and generalized additive models. Both for primary and secondary outcome, more events occurred in Group 1 compared with Group 2, while no significant difference was found in Group 3 with higher BP levels. Lower systolic BP variability showed better prognosis and higher CBF. The associations were more significant in patients with CAS ≥50%. BP variability exhibited a linear negative relationship with BP levels. In the early phase of AIS with hypertension and CAS, maintaining low blood pressure variability may be important to improve outcomes while low BP levels (SBP/DBP < 140/90 mm Hg) were harmful, especially in those patients with CAS ≥ 50%.

## 1 INTRODUCTION

Stroke is the third most common cause of death worldwide after ischemic heart disease and cancer. Ischemic stroke (IS) accounts for about 80% of all strokes.^1^ Carotid artery stenosis (CAS) is a major risk factor of IS and subsequent neurological dysfunction.^2^ CAS may decrease blood flow and cause brain hypoperfusion.^3^ Patients with severe carotid artery disease are at increased risk for recurrent stroke.^4^ Hypertensive patients with carotid artery stenosis often have high blood pressure (BP) during the acute phase of IS.^5^, ^6^ The management of high BP levels in acute phase is an unresolved issue in IS patients. High BP levels in the acute phase are related with a poor prognosis.^1^, ^7^, ^8^ However, low BP levels are also associated with poor prognosis in patients with IS as well.^9^ Our previous study confirmed that lower or higher BP levels in the early phase of IS were both correlated with increased risk of adverse outcomes.^10^

Recent studies have shown that for stroke patients, especially those with stroke and hypertension, BP variability is another important risk for recurrent stroke, cardiovascular disease, renal failure, and mortality.^11^ Higher long‐term visit‐to‐visit BP variability is associated with an increased risk of recurrent stroke, as well as major cardiovascular events and all‐cause death either in patients with hypertension or prior stroke.^12^, ^13^ Short‐term BP variability is also associated with neurological deteriorations in the acute phase of IS.^14^ Our previous study also demonstrated that higher BP variability within 7 days of onset was significantly correlated with the increased risk of recurrent stroke and composite cardiovascular events.^15^

Although there are many studies have suggested reasonable BP level and low BP variability were associated with good outcome, these studies did not focus on AIS patients with hypertension and CAS. It is an interesting question how to weigh BP level and BP variability in AIS patients with hypertension and CAS in the early stage. Unfortunately, no relevant study has been reported. Therefore, this study was aimed to investigate BP variability and proper BP level in early phase of acute ischemic stroke with hypertension and CAS.

## 2 METHODS

### 2.1 Study design and participants

**FIGURE 1:**
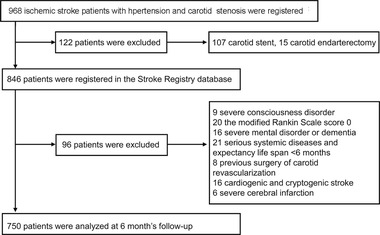
Flow chart of the patients enrolled in this study

We carried out a single‐center, prospective cohort study. A total of 968 IS patients with hypertension were registered in the Stroke Registry Database from January 2016 to December 2016. Of cases, 107 cases of carotid stenting and 15 of carotid endarterectomy were excluded. Patient were excluded with any one of the following reasons: severe disturbance of consciousness identified by the level of consciousness on National Institutes of Health Stroke Scale (NIHSS) score > 1; the mRS score 0 before stroke onset (20 patients); severe mental disorders or dementia; serious systemic diseases and life expected < 6 months; previous surgical carotid revascularization; cardiogenic and cryptogenic stroke; severe cerebral infarction (radiographic evidence of acute cerebral infarction, patients, a score of not less than 16 as per NIHSS on admission). Finally, 750 IS patients with hypertension and CAS were eligible for this study. In addition, in the 750 IS patients with hypertension and CAS, 404 patients with CAS < 50% did not need to undergo carotid revascularization, 283 patients with CAS > 50% but < 70% not undergo carotid revascularization due to the lack of surgical indications, and 63 patients with CAS ≥ 70% refused to undergo carotid revascularization since they were unable to pay for medical expenditure of revascularization surgery or fear of surgery even with severe stenosis. Study flow diagram of patients’ enrollment was shown in Figure 1.

### 2.2 Study measures

#### 2.2.1 Assessment of CAS and cerebral blood flow

CTA and ultrasonography were performed on the third day of admission. All CTA and ultrasound examinations were performed by trained cerebrovascular imaging examiners. The CTA scans used the Somatom Definition Flash CT system (Siemens Medical Systems, Germany). Contrast agent tracking was used in selected regions of interest at the aortic arch or carotid arteries to monitor the CT numbers. When the CT number exceeded 100 Hounsfield Units, the scan was automatically triggered with a delay of 4 s. We focused especially upon CAS based on the following causes: (1) extracranial CAS is the most important cause of large‐vessel stroke and is associated with 11.5% of all IS^16^; (2) internal carotid arteries (ICAs) carry about 70% of total cerebral blood flow (CBF) and the vertebral arteries (VAs) distribute about 30% of total CBF to the brainstem, cerebellum and occipital cortex.^17^ The diameter stenosis percentage was calculated using the measured diameter of the arterial lumen at the narrowest point (A) and the diameter of the original arterial lumen (B) as the diameter stenosis percentage (%) = (B−A)/B × 100%. The degree of stenosis of the internal carotid artery was evaluated using the following classification: none (0%), mild stenosis (< 50%), moderate stenosis (50–69%), severe stenosis (≥70%).

A color Doppler ultrasonography (CDUS) system, vivid E7 (GE, USA), was used for blood flow data acquisition of CBF. The hemodynamic parameters of the bilateral common carotid arteries, extracranial segment of the ICA and V1‐V3 segments of the VA were recorded, including peak systolic flow velocity (PSV), end‐diastolic flow velocity (EDV), and time‐averaged flow velocity (TAV). The intravascular flow volume (ml/min) was calculated by multiplying TAV with the cross‐sectional area (A) of the circular vessel according to the formula: flow volume = TAV × A = TAV × ([d/2]^2^ × π). The anterior circulation cerebral blood flow (CBF) volume was the sum of flow volumes of ICA both sides.^18^ The total CBF was measured for subsequent analysis.

#### 2.2.2 Blood pressure measurement

The treatments complied with 2013 American Heart Association/American Stroke Association (AHA/ASA) guidelines for the early management of patients with acute IS.^19^ After discharge, the treatments were consistent with 2013 AHA/ASA guidelines for the prevention of stroke in patients with stroke and transient ischemic attack.^19^ Supine BP was measured by trained nurses with a standard mercury sphygmomanometer^20^, ^21^, ^22^, ^23^, ^24^ (YUTU‐XJ11E; Medical Instrument Co, Shanghai, China) on the non‐paralyzed arm every 4 h for 3 days after stroke onset. Blood pressure was measured two consecutive readings between each measurement. At the end, the mean values of SBP and DBP measurements within 3 days of stroke onset were used as the levels of SBP and DBP in the early phase, respectively. The calibration examiners performed the standardized clinical examination for the mercury sphygmomanometer every 6 months. The authentication certificate is provided as Supplementary file.

#### 2.2.3 Blood pressure variability assessment

Blood pressure variability can be defined as the variation in blood pressure during a period of time (standard deviation [SD] or coefficient of variation).^25^ In our study, blood pressure variability was assessed by coefficients of variation. We calculated the coefficients of variation (CV [%]  =  SD × 100/mean value) during the IS acute stage.

### 2.3 Definitions

Hypertension was defined as a conventional BP of at least 140 mm Hg systolic or 90 mm Hg diastolic, or the use of antihypertensive drugs.^19^ Carotid artery stenosis was defined as the narrowing of the lumen of the carotid artery owing to carotid atherosclerotic plaque, arteritis, or fibromuscular dysplasia.^3^, ^26^ IS was defined as the occurrence of stroke identified by radiographic diagnosis (computed tomography [CT] or magnetic resonance imaging [MRI] scanning) and clinical diagnosis.^27^ The 750 cases included in this study all met the clinical diagnostic criteria for acute ischemic stroke, and were examined by CT or MRI. Cryptogenic stroke was defined as IS of undermined causes. Severe cerebral infarction was defined as IS patients with unstable vital signs in continuous rescue. The early phase of IS was defined as the time period within 3 days of symptom onset. Thrombolytic therapy was defined as intravenous administration of recombinant tissue‐type plasminogen activator (rt‐PA) within 4.5 h of symptom onset^28^ or catheter‐directed thrombolysis using rt‐PA within 12 h of symptom onset. Cardiac death was defined as death due to lethal cardiac arrhythmias, congestive heart failure, myocardial infarction, sudden cardiac death, and other known vascular causes.

All the patients had been taking antihypertensive agent and controlled well before admission. They continued taking drugs as normal after admission to control hypertension. Based on the average BP levels in the 3 days after stroke onset, participants were categorized as controlled BP group (Group 1: SBP < 140 mm Hg and DBP < 90 mm Hg), mild hypertension group (Group 2: SBP :140–159 mm Hg and or DBP: 90–99 mm Hg), and moderate to severe hypertension group (Group 3: SBP ≥160 mm Hg and/or DBP ≥100 mm Hg) according to the Guidelines for Hypertension.^29^ CV of SBP or DBP and global CBF were grouped into tertiles.

### 2.4 Patient follow‐up and outcome assessment

All participants were followed up in 6 months by trained neurologists. Primary outcome was recurrent stroke ([fatal and non‐fatal] ischemic and hemorrhagic stroke) at 6 months.^30^ Secondary outcome included all‐cause death and the composite cardiovascular events (recurrent stroke, non‐fatal myocardial infarction) at 6 months.^30^ After discharge, patients received regular review by specialist during the follow‐up period. The treatment for hypertension and stroke was according to the corresponding guidelines.^19^

### 2.5 Statistical analysis

All statistical analysis was performed with SPSS.V24.0 software packages. Continuous variables were presented as mean (SD) and were compared using the Student *t* test or one‐way ANOVA. Categorical variables were expressed as frequency (percentage) and were compared using χ^2^ test. Pearson correlation was performed for the association between BP levels and BP variability. To explore nonlinear relations between BP variability and global CBF, generalized additive models (GAMs) were constructed by using R statistical software (version 4.0.2). Kaplan‐Meier (KM) curves were plotted to illustrate event outcomes among groups, and the statistical significance was determined by the log‐rank test. Associations between parameters (BP levels, CV of BP, and CBF) and primary and secondary outcomes were analyzed using Cox proportional hazard analysis. Univariate logistic regression was carried out for each risk factor provided on baseline characteristics, *p* values were more than 0.05 except for SBP and CV of SBP. Multivariable model induced traditional cardiovascular risk factors: age, male, body mass index, smoking, alcohol, diabetes mellitus, coronary artery disease, congestive heart failure, chronic kidney disease, NIHSS according to the relevant literatures,^15^, ^31^ and standard deviation of SBP was also included as an independent variable adjusted for the BP levels when patients were grouped according to the BP levels. *p* < 0.05 was considered statistically significant.

### 2.6 Standard protocol approvals and patient consents

The study was approved by the ethic committee of Lianyungang Hospital. Written informed consent was obtained from each patient or his/her proxy.

## 3 RESULTS

### 3.1 Demographics and clinical characteristics

Of the 750 patients with first‐ever IS and CAS, 404 (53.9%) had mild stenosis, 131 (17.5%) had moderate stenosis, 111 (14.8%) had severe stenosis and 104 (13.9%) had complete occlusion, respectively. Baseline characteristics of 750 patients based on groups of BP levels in the early phase were shown in Table 1. Besides, antihypertensive medications were classified as ACE inhibitor (ACEI) or angiotensin receptor blocker (ARB), calcium channel blockers (CCB), beta blockers (BB) and diuretics in our study. Most patients took two or more antihypertensive drugs. In hospital, 21 patients in Group 3 with blood pressure ≥200/110 mm Hg within 48 h of onset additionally received intravenous sodium nitroprusside or nitroglycerin to maintain their blood pressure lower than 180/100 mm Hg. Except for this, there were no obvious differences on antihypertensive medication among groups before admission and in‐hospital/after discharge. The detail information is shown in Table S1.

**TABLE 1:** Baseline characteristics of the study population based on blood pressure levels in the early phase of ischemic stroke

Characteristics	Group 1 (*N* = 262)	Group 2 (*N* = 230)	Group 3 (*N* = 258)	*P* value
Age (years), mean (SD)	64.9 (12.38)	65.2 (12.45)	64.5 (12.53)	0.81
Male, *n* (%)	160 (61.1)	144 (62.6)	153 (59.3)	0.755
BMI (kg/m^2^), mean (SD)	24.8 (3.14)	25.3 (3.22)	25 (3.51)	0.264
SBP, mean (SD), mm Hg	127 (6.5)	149 (7.4)	173 (7.4)	<0.001
DBP, mean (SD), mm Hg	73 (5.2)	84 (5.7)	97 (5.7)	<0.001
Admission SBP (mm Hg), median (IQR)	140 (80‐210)	146 (94‐238)	148(100‐260)	<0.001
Admission DBP (mm Hg), median (IQR)	80 (60‐120)	82 (60‐126)	85 (60‐152)	0.036
Admission NIHSS, median (IQR)	2 (1‐5)	1 (1‐4)	2 (1‐5)	0.09
CV of SBP, mean (SD)	15.5 (4.9)	13.6 (3.6)	11.9 (3.0)	<0.001
CV of DBP, mean (SD)	15.7(5.1)	13.4 (5.5)	13.9(5.0)	<0.001
Smoking, *n* (%)	116 (44.3)	87 (37.8)	102 (39.5)	0.314
Alcohol, *n* (%)	97 (37.0)	72 (31.3)	83 (32.2)	0.34
**Medical history**				
Diabetes mellitus, *n* (%)	71 (27.1)	52 (22.6)	56 (21.7)	0.306
Coronary artery disease, *n* (%)	29 (11.1)	21 (9.1)	21 (8.1)	0.51
Congestive heart failure, *n* (%)	4 (1.5)	2 (0.9)	4 (1.6)	0.762
Chronic kidney disease, *n* (%)	0	2 (0.9)	3 (1.2)	0.239
**Stroke features**				
mRS score, mean (SD)	2.8 (1.2)	2.6 (1.2)	2.5 (1.3)	0.017
Thrombolytic therapy, *n* (%)	6 (2.3)	4 (1.7)	1 (0.4)	0.18
**Treatment after discharge**				
Glucose‐lowering agents, *n* (%)	62 (23.7)	52 (22.6)	49 (19.0)	0.403
Lipid‐lowering agents, *n* (%)	261 (99.6)	227 (98.7)	255 (98.8)	0.509
Antiplatelet agents, *n* (%)	258 (98.5)	224 (97.4)	251 (97.3)	0.606
Anticoagulants, *n* (%)	21 (8.0)	15 (6.5)	13 (5.0)	0.389

Group 1: SBP < 140 mm Hg and DBP < 90 mm Hg, Group 2: SBP :140‐159 and/ or DBP: 90–99 mm Hg; Group 3: SBP ≥160 and/or DBP ≥100 mm Hg. Data are mean (SD) or median (IQR) for continuous variables, and *n* (%) for categorical variables.

*Abbreviations*: NIHSS, National Institute of Health Stroke Scale, SBP, systolic blood pressure, DBP, diastolic blood pressure. CV, coefficient of variation of SBP.

### 3.2 More events in group with lower BP levels, while no significant difference in group with higher BP levels

**FIGURE 2:**
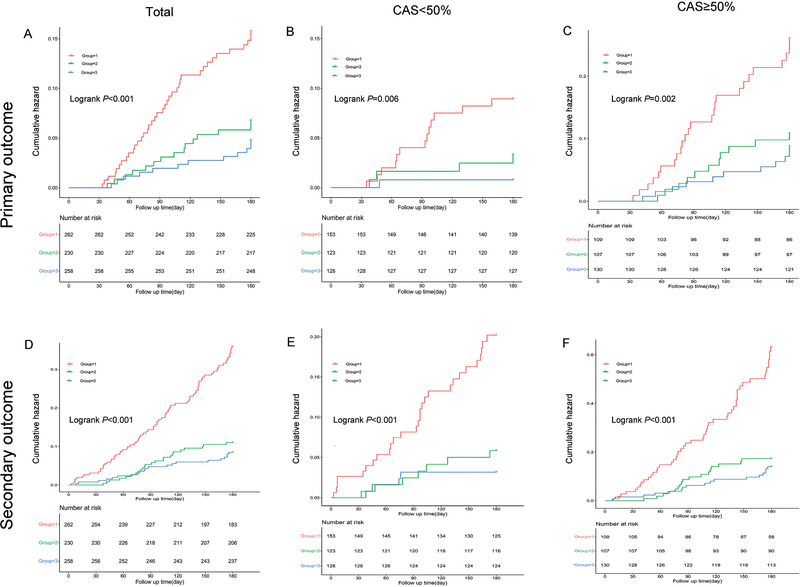
KM cumulative hazard curves demonstrating the association of the BP levels with the primary and secondary outcome. KM curves for the association of BP with the primary outcome in total (A), in group with CAS < 50% (B), and in group with CAS ≥50% (C); KM cumulative hazard curves for the association of BP with the secondary outcome in total (D), in the group with CAS < 50% (E), and in the group with CAS ≥ 50% (F). The patients were divided into three groups according to BP levels. Group1: SBP < 140 mm Hg and DBP < 90 mm Hg, Group 2: SBP :140‐159 and/ or DBP: 90–99 mm Hg; Group 3: SBP ≥160 and/or DBP ≥100 mm Hg

Univariate Cox regression analysis for primary and secondary outcomes are shown in Table S2. When SBP < 140 mm Hg or SBP < 160 mm Hg, increased SBP was a significant protective factor for primary and secondary outcomes. However, when SBP≥160 mm Hg, the hazard ratio was 1.011 with no statistical significance. The relationships between BP and primary/secondary outcomes by multivariate Cox regression analysis were shown in Table 2. Patients with lowest BP levels (SBP < 140 and DBP < 90 mm Hg) had a higher risk of recurrent stroke (HR = 2.66, 95%CI: 1.38–5.16, *p* < 0.01), all‐cause death and the composite cardiovascular events (HR = 3.93, 95%CI: 2.13–5.40, *p *< 0.001) at 6 months. However, there were no significant association with patients in higher BP levels group (SBP/DBP ≥160/100 mm Hg) and incidence of primary outcome (*p *= 0.501) or secondary outcome (*p *= 0.715). Similar associations were found both in group with CAS < 50% and in group with CAS ≥50%. *KM* curves (Figure 2) illustrated that the occurrence percentages of the primary outcome and secondary outcome were higher in the lowest BP group compared with the other BP groups (both *p *< 0.001).

**TABLE 2:** Association of BP levels in the early phase and primary and secondary outcomes at 6 months

	Primary outcome			Secondary outcome		
Groups of BP	Events/patients (%)	Raw HR (95%CI)	Adjusted HR (95%CI)	Events/patients (%)	Raw HR (95%CI)	Adjusted HR (95%CI)
**Total**						
Group 1	36/262 (13.7)	2.76 (1.44‐5.31)^*^ ^*^	2.48 (1.35‐4.55) ^*^ ^*^	78/262 (29.8)	3.22 (2.04‐5.09)***	3.48 (2.19‐5.53)^***^
Group 2	12/230 (5.2)	Ref	Ref	24/230 (10.4)	Ref	Ref
Group 3	10/258 (3.9)	0.73 (0.32‐1.70)	0.78 (0.36‐1.69)	21/258 (8.1)	0.77 (0.43‐1.38)	0.84 (0.47‐1.53)
**CAS < 50%**						
Group 1	15/153 (9.8)	4.17 (1.21‐14.42)^*^	2.79 (0.89‐8.74)	28/153 (18.3)	3.44 (1.50‐7.88)^**^	3.42 (1.47‐7.95)^**^
Group 2	3/123 (2.4)	Ref	Ref	7/123 (5.7)	Ref	Ref
Group 3	1/128 (0.8)	0.32 (0.03‐3.07)	0.18 (0.02‐1.65)	4/128 (3.1)	0.55 (0.16‐1.88)	0.52 (0.15‐1.88)
**CAS ≥50%**						
Group 1	21/109 (19.3)	2.46 (1.13‐5.37)^*^	2.88 (1.38‐6.03) ^*^	50/109 (45.9)	3.53 (2.04‐6.12)^***^	3.74 (2.12‐6.59) ^***^
Group 2	9/107 (8.4)	Ref	Ref	17/107 (15.9)	Ref	Ref
Group 3	9/130 (6.9)	0.63 (0.32‐2.01)	0.84 (0.36‐2.00)	17/130 (13.1)	0.80 (0.41‐1.57)	0.77 (0.38‐1.54)

The patients in the study were divided into three groups according to BP levels. Group 1: SBP < 140 mm Hg and DBP < 90 mm Hg, Group 2: SBP :140‐159 and/ or DBP: 90–99 mm Hg; Group 3: SBP ≥160 and/or DBP ≥100 mm Hg. Age, male, BMI, SBP, standard deviation of SBP, smoking, alcohol, diabetes mellitus, coronary artery disease, congestive heart failure, chronic kidney disease, NIHSS were induced for adjustment.

*Abbreviations*: HR, hazard ratio; CI, confidence interval; NIHSS, National Institutes of Health Stroke Scale.

* *p *< .05.

** *p *< .01.

*** *p *< .001.

### 3.3 Low BP variability was associated with good prognosis

**FIGURE 3:**
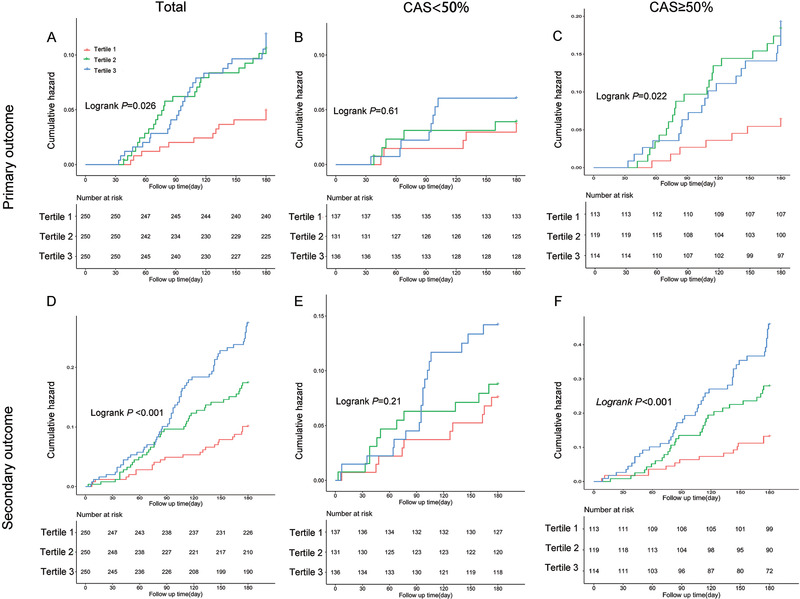
KM cumulative hazard curves demonstrating the association of SBP variability with the primary and secondary outcome. KM cumulative hazard curves for the association of CV of SBP with the primary outcome in total (A), in group with CAS < 50% (B), and in group with CAS ≥50% (C); KM cum hazard curves for the association of CV of SBP with the secondary outcome in total (D), in the group with CAS < 50% (E), and in the group with CAS ≥ 50% (F). All patients were divided into tertile groups according to CV of SBP. Tertile 1 ≤ 11.85, Tertile 2: 11.86‐14.96, Tertile 3 ≥14.97

BP variability was also an important risk factor for prognosis. Next, we analyzed the relationship between BP variability and prognosis. Cox regression showed no significant difference between DBP variability and prognosis (Table S3 and Figure S1). Figure 3 and Table 3 in the showed patients with the lowest SBP variability had the best prognosis, including primary outcome and secondary outcome. After stratified with CAS, the association was only found in group with CAS ≥50% but not in group with CAS < 50%.

**TABLE 3:** Association of CV of SBP in the early phase and primary and secondary outcomes at 6 months

	Primary outcome			Secondary outcome		
Groups	Events/patients (%)	Raw HR (95%CI)	Adjusted HR (95%CI)	Events/patients (%)	Raw HR (95%CI)	Adjusted HR (95%CI)
**Total**						
Tertile 1 (≤11.85)	12/250 (4.8)	0.46 (0.23‐0.92)^*^	0,42 (0.21‐0.85)^*^	24/250 (9.6)	0.37 (0.23‐0.59)^***^	0.35 (0.22‐0.57)^***^
Tertile 2 (11.86‐14.96)	21/250 (8.4)	0.83 (0.46‐1.49)	0.79 (0.44‐1.42)	40/250 (16.0)	0.64 (0.43‐0.96)^*^	0.61 (0.41‐0.92)^*^
Tertile 3 (≥14.97)	25/250 (10.0)	Ref	Ref	59/250 (23.6)	Ref	Ref
*P* for trend		0.029	0.018		<0.001	<0.001
**CAS < 50%**						
Tertile 1 (≤11.85)	6/137 (4.4)	0.74 (0.26‐2.12)	0.66 (0.22‐2.00)	10/137 (7.3)	0.53 (0.25‐1.16)	0.58 (0.26‐1.30)
Tertile 2 (11.86‐14.96)	5/131 (3.8)	0.65 (0.21‐1.99)	0.58 (0.19‐1.81)	11/131 (8.4)	0.63 (0.30‐1.33)	0.66 (0.31‐1.42)
Tertile 3 (≥14.97)	8/136 (5.9)	Ref	Ref	18/136 (13.2)	Ref	Ref
*P* for trend		0.565	0.569		0.103	0.155
**CAS ≥50%**						
Tertile 1 (≤11.85)	6/113 (5.3)	0.33 (0.13‐0.85)^*^	0.31 (0.12‐0.81)^*^	14/113 (12.4)		0.27 (0.15‐0.51)^***^
Tertile 2 (11.86‐14.96)	16/119 (13.4)	0.88 (0.44‐1.74)	0.79 (0.39‐1.60)	29/119 (24.4)		0.54 (0.33‐0.89)^*^
Tertile 3 (≥14.97)	17/114 (14.9)	Ref	Ref	41/114 (36.0)	Ref	Ref
*P* for trend		0.022	0.014		<0.001	<0.001

All patients were divided into tertile groups according to CV of SBP. Age, male, BMI, smoking, alcohol, diabetes mellitus, coronary artery disease, congestive heart failure, chronic kidney disease, NIHSS were induced for adjustment.

*Abbreviations*: HR, hazard ratio; CI, confidence interval; NIHSS, National Institutes of Health Stroke Scale.

* *p *< .05.

** *p *< .01.

*** *p *< .001.

### 3.4 BP variability was negatively associated with CBF volume

**FIGURE 4:**
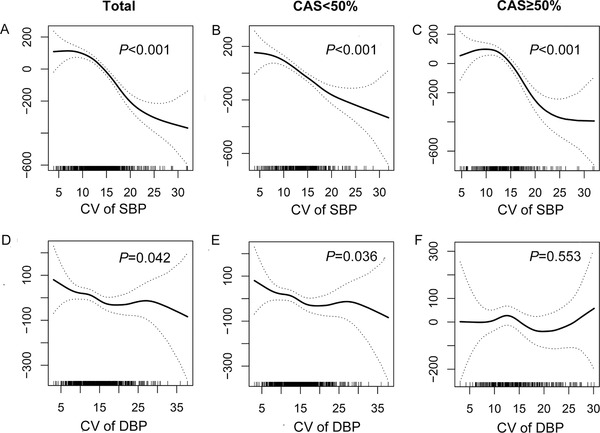
GAM plots of the global CBF and BP variability. GAM plots shown are between the global CBF and (A) CV of SBP, (B) CV of SBP with CAS < 50% (C) CV of SBP with CAS ≥50% (D) CV of DBP, (E) CV of DBP with CAS < 50%, (F) CV of DBP with CAS ≥50%

CBF is necessary for conservation of brain function, and hypoperfusion might cause brain infarction, which in turn leads to a poor prognosis. Furthermore, we determined the association of BP variation with CBF. Pearson correlation analysis and curve fitting showed an overall negative correlation between SBP variability and global CBF (*p *< 0.001). In ischemic stroke patients with CAS ≥50%, a negative linear correlation was observed between SBP variability and CBF compared with ischemic stroke patients with CAS < 50%. The relation between BP variability and global CBF was also shown graphically as GAM plots in Figure 4 (*p *< 0.001).

### 3.5 CBF levels were associated with prognosis

Table 4 showed the association between CBF levels and prognosis. Higher global CBF in the early phase was associated with better primary and secondary outcomes at 6 months. Patients in the lowest CBF group (< 457 ml/min) showed the worst primary outcome comparing to other groups. Patients with CBF < 730 ml/min were associated with poor secondary outcome. After stratified with CAS, the association still presented. Meanwhile, KM curves also revealed that the primary outcome and secondary outcome were significantly worse in the lowest CBF group compared with the highest CBF group (*p *< 0.001) (Figure S2 in the Supplement).

**TABLE 4:** The relationship between global CBF and primary outcome and secondary outcome

	Primary outcome			Secondary outcome		
Groups	Events/patients (%)	Raw HR (95%CI)	Adjusted HR (95%CI)	Events/patients (%)	Raw HR (95%CI)	Adjusted HR (95%CI)
**Total**						
Tertile 1 (< 457)	36/250 (14.0)	5.49 (2.44‐12.33)^***^	5.48 (2.42‐12.41)^***^	75/250 (30.0)	11.09 (5.35‐22.99)^***^	12.13 (5.83‐25.26)^***^
Tertile 2 (457‐729)	15/250 (6.0)	2.21 (0.91‐5.42)	2.10 (0.86‐5.17)	40/250 (16.0)	5.36 (2.51‐11.44)^***^	5.28 (2.47‐11.28)^***^
Tertile 3 (≥730)	7/250 (2.8)	Ref	Ref	8/250 (3.2)	Ref	Ref
*P* for trend		<0.001	<0.001		<0.001	<0.001
**CAS < 50%**						
Tertile 1 (< 457)	13/141 (9.2)	11.97 (1.52‐90.71)^*^	12.47 (1.61‐96.39)^*^	26/141 (18.4)	12.35 (2.93‐52.02)^**^	12.98 (3.04‐55.52)^**^
Tertile 2 (457‐729)	5/139 (3.6)	4.60 (0.54‐39.3)	4.51 (0.52‐38.94)	11/129 (8.5)	5.10 (1.13‐23.01)^*^	4.84 (1.07‐21.91)^*^
Tertile 3 (≥730)	1/124 (8.1)	Ref	Ref	2/124 (1.6)	Ref	Ref
*P* for trend		0.003	0.003		<0.001	<0.001
**CAS ≥ 50%**						
Tertile 1 (< 457)	23/109 (21.1)	4.90 (2.00‐12.04)^**^	5.45 (2.18‐13.66)^***^	49/109 (45.0)	12.60 (5.40‐29.40)^***^	14.99 (6.37‐35.25)^***^
Tertile 2 (457‐729)	10/111 (9.0)	1.95 (0.71‐5.37)	1.87 (0.67‐5.20)	29/111 (26.1)	6.14 (2.55‐14.79)^***^	6.07 (2.51‐14.70)^***^
Tertile 3 (≥730)	6/126 (4.8)	Ref	Ref	6/126 (4.8)	Ref	Ref
*P* for trend		<0.001	<0.001		<0.001	<0.001

All patients were divided into tertile groups according to global CBF. Age, male, BMI, smoking, alcohol, diabetes mellitus, coronary artery disease, congestive heart failure, chronic kidney disease, NIHSS were induced for adjustment. CBF, Cerebral blood flow; HR, hazard ratio; CI, confidence interval; NIHSS, National Institutes of Health Stroke Scale.

* *p *< .05.

** *p *< .01.

**** *p *< .001.

### 3.6 BP levels were negatively associated with BP variability

**FIGURE 5:**
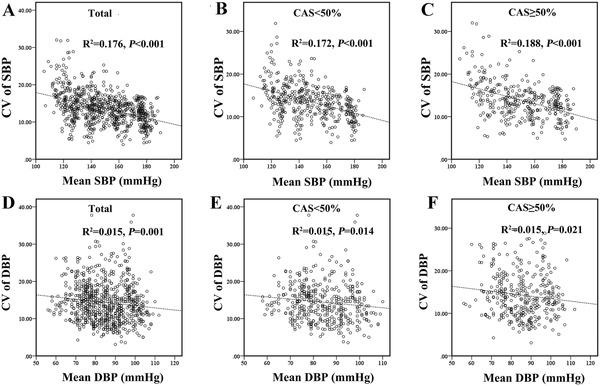
The correlation between BP levels and CV of BP. The correlation between SBP levels and variability in total (A), in group with CAS < 50% (B), and in group with CAS ≥50% (C); the correlation between DBP levels and variability in total (D), in the group with CAS < 50% (E), and in the group with CAS ≥50% (F)

Low BP levels were associated with a poor prognosis, while low BP variability was associated with a good prognosis in our study. Then, what's the relation between BP levels and its variation? The correlation between BP levels and BP variability was evaluated by Pearson correlation analysis. Pearson correlation analysis revealed there was a negative correlation between BP groups and SBP variability and DBP variability, respectively. In patients with CAS ≥ 50%, there was a still negative linear correlation was observed between BP variability and SBP (*p *< 0.001) and DBP (*p *= 0.021) (Figure 5). In other words, when BP was maintained at relatively higher level, BP variability was generally low in the study population.

## 4 DISCUSSION

In the present study, we demonstrated that in IS patients with hypertension and CAS, lower BP levels (< 140/90 mm Hg) led to a poor prognosis, while higher BP levels (> 160/100 mm Hg) did not show better prognosis, and BP levels was negatively correlated with BP variability. On the other hand, low BP variability in the early phase (within 3 days after onset) was associated with adequate cerebral perfusion and then reduced risks of primary and secondary outcomes at 6 months, particularly in those patients with CAS ≥50%. So, maintaining low blood pressure variability may be important to improve outcomes while low BP levels (SBP/DBP < 140/90 mm Hg) were harmful, especially in those patients with CAS ≥ 50%.

Hypertension in the acute phase is associated with a poor prognosis but lowering blood pressure might also be harmful. The Scandinavian Candesartan Acute Stroke Trial (SCAST) indicated that blood pressure lowering treatment in acute stroke was associated with an increased risk of stroke progression and poor functional outcome^32^ . The International Stroke Trial study found a U‐shaped relationship between BP and outcome, indicating that both high and low BP levels were prognostic factors for poor outcome in IS patients.^7^ In our study, more events were found in group with the lower BP (SBP < 140 mm Hg and DBP < 90 mm Hg), especially in patients with CAS ≥50%, while there was no obvious association between the highest BP group (SBP ≥160 and/or DBP ≥100 mm Hg) and poor prognosis. Owing to the low number of patients with severe hypertension in the highest BP group in this study, the result might not be generalized. For hypertensive patients with CAS, lower BP levels, which were close to the “ideal blood pressure level”, were not good for both primary outcome and secondary outcome in our study.

Furthermore, in the present study, low BP variability in the early phase, measured by CV, was significantly associated with good prognosis. The trend was more obvious in these patients with CAS ≥50%. Our findings are consistent with some previous studies. Bum Joon Kim and coworkers demonstrated that increased BP variability was strongly correlated with the risk of recurrent stroke in patients with hypertension and a history of previous stroke or TIA.^33^ In the VALUE trial, higher SBP variability was associated with increased risk of cardiovascular events in patients with hypertension.^13^ The PRoFESS study also found that in patients with recent IS, higher BP variability was associated with an increased risk of recurrent ischemic stroke, major cardiovascular events, and all‐cause death.^12^

Inadequate cerebral perfusion and impaired cerebral autoregulation in the early phase of stoke increased cardiovascular complications, and secondary brain injury. These conditions in turn might cause poor outcome after acute IS. We found lower BP variability was correlated with higher global CBF, and the higher global CBF were more beneficial to prognosis in our study. Patients with lower CV of BP maintained a relatively high global CBF, which was in turn advantageous for prognosis.

The relationship between BP variability and prognosis was more significant in patients with CAS ≥50%, while no statistically significant differences in patients with CAS < 50%. In normal condition, cerebral autoregulation maintains blood flow to the brain and regulates cerebral perfusion pressure changes over a wide range. BP level and BP variability are major factors affecting cerebral perfusion. In the IS population with hypertension and mild CAS or none, the collateral circulation would compensate for the influence of blood pressure fluctuations on brain to prevent cerebral hypoperfusion. The circle of Willis is considered to be the primary collateral flow route which can supplement the affected brain tissue area with blood.^34^, ^35^ When the degree of CAS exceeded a critical threshold (CAS≥50%) or the collateral circulation couldn't compensate for the reduction of CBF, the incidence of IS would increase in this situation.

Therefore, for IS patients with hypertension and CAS ≥50% in the early phase of stroke, a lower blood pressure variability and higher BP levels can compensate for inadequate collateral circulation by maintaining a relatively high cerebral blood flow. The relatively stable and high cerebral perfusion reduces stroke recurrence and improves the prognosis of stroke. Low BP variability and maintaining reasonable BP levels (SBP 140–160 and DBP 90–100 mm Hg) were more beneficial in the present study.

This study has limitations. First, we did not include flow volumes of vertebral arteries and only calculated stenosis of carotid arteries, the advantage bias might exist. Second, the sample size of this study was relatively small; however, it was a real clinical practice, in which strict inclusion criteria were used, data were reasonably analyzed, and conclusions was carefully scrutinized.

As a result, our study might deliver useful recommendation for BP management in the early phase of IS patients in this group of patients with CAS.

## 5 CONCLUSIONS

In our study, more events were found in group with the lower BP (SBP < 140 mm Hg and DBP < 90 mm Hg), while there was no obvious association between the highest BP group (SBP ≥160 and/or DBP ≥100 mm Hg) and poor prognosis. Owing to the small number of patients with severe hypertension (SBP≥180 mm Hg and/or DBP ≥110 mm Hg) in our study, this result regarding to extremely high blood pressure levels might not be generalized. It could be concluded that maintaining low BP variability and reasonable BP levels (SBP140 ∼160 and DBP 90 ∼ 100 mm Hg) were important to reduce the risk of recurrent stroke, all‐cause death and the composite cardiovascular events in the early phase of IS with hypertension and CAS.

## CONFLICT OF INTEREST

None.

## AUTHOR CONTRIBUTIONS

Mingli He and Yibo Wang conceived and designed the research, Taotao Wu, Mingyu Wang, Ru Yang, Bo Zhang, Bingchao Xu, Xiaobing He, Guanghui Zhang, Xiaoqin Niu, Zaipo Li, Bei Wang and Bei Xu acquired the data, Bing Cui, Jin'e Wang, Xiao Xiao analyzed and interpreted the data, Bing Cui, Jin'e Wang, Xiao Xiao and Taotao Wu performed statistical analysis, Mingli He and Yibo Wang handled funding and supervision, Bing Cui and Jin'e Wang drafted the manuscript, Rutai Hui made critical revision of the manuscript for important intellectual content.

## Supporting information



Supporting information
