# Supplementary material for: Focus on blood pressure levels and variability in the early phase of acute ischemic stroke with hypertension and carotid stenosis
Source: J Clin Hypertens (Greenwich). 2021 Nov 16;23(12):2089–99. doi: 10.1111/jch.14385 (PMC8696233; doi:10.1111/jch.14385)
Supplement: Supplementary file 1 — Supporting information [file JCH-23-2089-s001.docx]

**Supplemental material**

**eTable 1.** Antihypertensive medication before and during in-hospital and after discharge.

**eTable 2.** Univariate Cox regression between SBP and primary and secondary outcomes.

**eTable 3.** Association of CV of DBP in the early phase and primary and secondary outcomes at 6 months.

**eFigure 1**. KM cumulative hazard curves demonstrating the association between the CV of DBP groups and the primary and secondary outcome.

**eFigure 2**. KM cumulative hazard curves for the relationships between global CBF and primary and secondary outcome.

**eTable 1 Antihypertensive medication before and during in-hospital and after discharge.**

|  |  | **Group 1** | **Group 2** | **Group 3** | ***P* value** |
| --- | --- | --- | --- | --- | --- |
| N |  | 262 | 230 | 258 |  |
| **Before admission** |  |  |  |  |  |
| CCB, n (%) |  | 32 (12.21) | 43 (18.70) | 37 (14.34) | 0.125 |
| ACEI or ARB, n (%) |  | 47 (17.94) | 31 (13.48) | 39 (15.12) | 0.383 |
| BB, n (%) |  | 10 (3.82) | 12 (5.22) | 7 (2.710) | 0.359 |
| ACEI /ARB+ Diuretics |  | 68 (25.95) | 50 (21.74) | 71 (27.52) | 0.384 |
| ACEI /ARB+CCB, n (%) |  | 51 (19.47) | 31 (13.48) | 48 (18.60) | 0.173 |
| CCB+BB, n (%) |  | 29 (11.07) | 37 (16.09) | 23 (8.91) | 0.090 |
| BB+ Diuretics, n (%) |  | 19 (7.257) | 21 (9.13) | 24 (9.30) | 0.654 |
| CCB+ Diuretics, n (%) |  | 6 (2.29) | 5 (2.17) | 9 (3.49) | 0.598 |
|  | |  |  |  |  |
| **Intravenous injection within 48 hours of onset*** |  | 0 | 0 | 21(8.14) | <0.001 |
| **In-hospital and after discharge** |  |  |  |  |  |
| CCB, n (%) |  | 51 (19.47) | 39 (16.96) | 40 (15.50) | 0.546 |
| ACEI or ARB, n (%) |  | 40 (15.27) | 42 (18.26) | 58 (22.48) | 0.083 |
| BB, n (%) |  | 7 (2.67) | 5 (2.17) | 11 (4.26) | 0.369 |
| ACEI /ARB+ Diuretics, n (%) |  | 71 (27.10) | 60 (26.09) | 52 (20.16) | 0.179 |
| ACEI /ARB+CCB, n (%) |  | 50 (19.08) | 48 (20.87) | 58 (22.48) | 0.570 |
| CCB+BB, n (%) |  | 19 (7.25) | 14 (6.09) | 17 (6.59) | 0.869 |
| BB+ Diuretics, n (%) |  | 23 (8.77) | 19 (8.26) | 15 (5.81) | 0.512 |
| CCB+ Diuretics, n (%) |  | 1 (0.38) | 3 (1.30) | 7 (2.71) | 0.456 |

The patients in the study were divided into three groups according to BP levels. Group 1: SBP<140 mmHg and DBP<90 mmHg, Group 2: SBP :140-159 and/ or DBP: 90-99 mmHg; Group 3: SBP ≥160 and/or DBP ≥100 mmHg. ACEI, ACE inhibitor; ARB, angiotensin receptor blocker; CCB, calcium channel blockers; BB, beta blockers.

*****: In hospital, 21 patients in Group 3 with blood pressure ≥200/110mmHg within 48 hours of onset additionally received intravenous sodium nitroprusside or nitroglycerin to maintain their blood pressure lower than 180/100mmHg.

**eTable2.** Univariate Cox regression between SBP and primary and secondary outcomes.

| **SBP** | **Primary outcome** | | |  | **Secondary outcome** | | |
| --- | --- | --- | --- | --- | --- | --- | --- |
|  | ***P* value** | **HR** | **95% CI** |  | ***P* value** | **HR** | **95% CI** |
| SBP<140 mmHg | 0.047 | 0.959 | 0.921-0.999 |  | <0.001 | 0.949 | 0.922-0.977 |
| SBP≥ 140 mmHg | 0.346 | 0.987 | 0.960-1.015 |  | 0.387 | 0.990 | 0.969-1.012 |
| SBP<160 mmHg | 0.002 | 0.968 | 0.948-0.988 |  | <0.001 | 0.953 | 0.938-0.968 |
| SBP≥ 160 mmHg | 0.792 | 1.011 | 0.930-1.099 |  | 0.914 | 0.997 | 0.938-1.059 |

**eTable 3. Association of CV of DBP in the early phase and primary and secondary outcomes at 6 months**

| **Groups** |  | **Primary outcome** | | |  | **Secondary outcome** | | |
| --- | --- | --- | --- | --- | --- | --- | --- | --- |
|  |  | **Events /patients (%)** | **HR** | ***P* value** |  | **Events /patients (%)** | **HR** | ***P* value** |
|  |  |  | **(95%CI)** |  |  |  | **(95%CI)** |  |
| **Total** |  |  |  |  |  |  |  |  |
| Tertile 1 (≤11.59) | | 17/250 (6.8) | 0.81 (0.43-1.54) |  |  | 44/250 (17.6) | 1.16 (0.76-1.79 |  |
| Tertile 2 (11.60-15.89) | | 20/250 (8.0) | 0.96 (0.52-1.76) |  |  | 41/250 (16.4) | 1.06 (0.68-1.64) |  |
| Tertile 3 (≥15.90) | | 21/250 (8.4) | Ref | - |  | 38/250 (15.2) | Ref | - |
| *P* for trend | |  |  | 0.538 |  |  |  | 0.499 |
| **CAS<50%** | |  |  |  |  |  |  |  |
| Tertile 1 (≤11.59) | | 8/131 (6.1) | 1.09 (0.41-2.90) |  |  | 15/131 (11.5) | 1.19 (0.58-2.47) |  |
| Tertile 2 (11.60-15.89) | | 3/132 (2.3) | 0.39 (0.10-1.48) |  |  | 10/132 (7.6) | 0.75 (0.33-1.69) |  |
| Tertile 3 (≥15.90) | | 8/142 (5.7) | Ref | - |  | 14/141 (9.9) | Ref | - |
| *P* for trend | |  |  | 0.997 |  |  |  | 0.709 |
| **CAS ≥ 50%** | |  |  |  |  |  |  |  |
| Tertile 1 (≤11.59) | | 9/119 (7.6) | 0.63 (0.27-1.46) |  |  | 29/119 (24.4) | 1.09 (0.64-1.86) |  |
| Tertile 2 (11.60-15.89) | | 17/118 (14.4) | 1.25 (0.61-2.57) |  |  | 31/118 (26.3) | 1.18 (0.70-1.99) |  |
| Tertile 3 (≥15.90) | | 13/109 (11.9) | Ref | - |  | 24/109 (22.0) | Ref | - |
| *P* for trend | |  |  | 0.361 |  |  |  | 0.732 |

All patients were divided into tertile groups according to CV of DBP. Age, male, BMI, smoking, alcohol, diabetes mellitus, coronary artery disease, congestive heart failure, chronic kidney disease, NIHSS were induced for adjustment. HR, hazard ratio; CI, confidence interval; NIHSS, National Institutes of Health Stroke Scale. ^1^ *P*<0.05,^2^ *P*<0.01, ^3^ *P*<0.001.


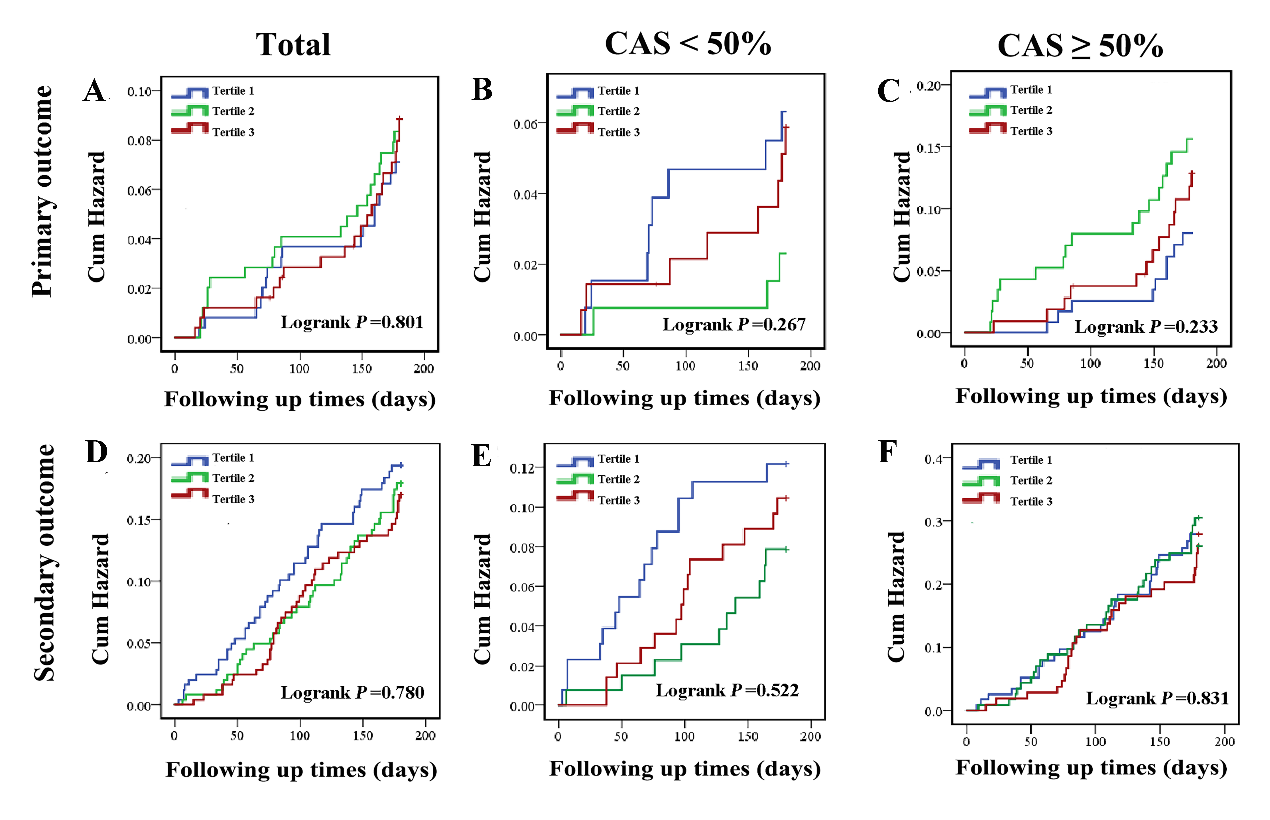


**eFigure 1.** **KM cumulative hazard curves demonstrating the association between the** **CV of DBP groups and the primary and secondary outcome.**

KM cum hazard curves for the association of CV of DBP in total (A), in group with CAS<50% (B), and in group with CAS ≥50% (C); KM cum hazard curves for the association of CV of DBP with the secondary outcome in total (D), in the group with CAS<50% (E), and in the group with CAS ≥ 50% (F).


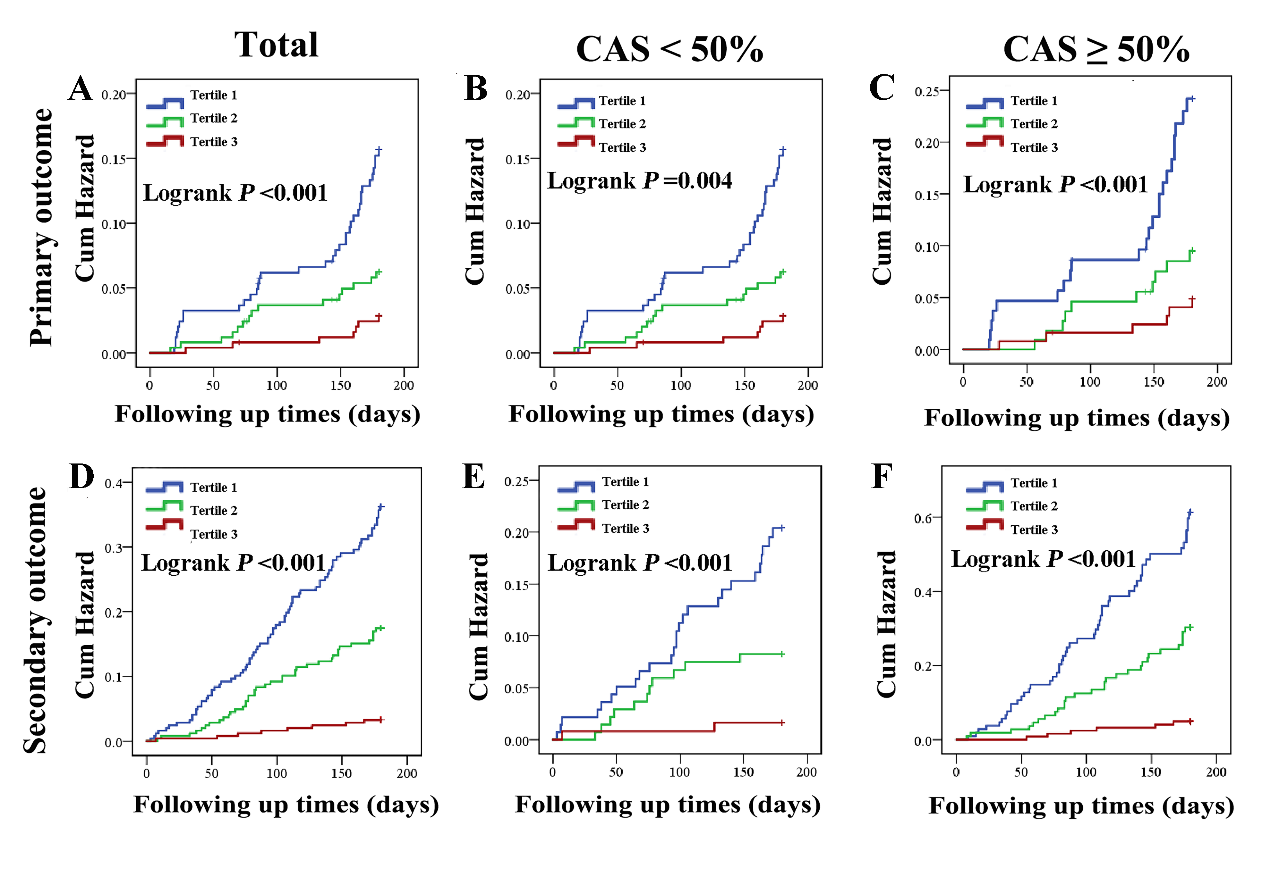


**eFigure 2.** **KM cumulative hazard curves for the relationships between** **global CBF and primary and secondary outcome.**

KM cum hazard curves for the association of global CBF in total (A), in group with CAS<50% (B), and in group with CAS ≥50% (C); KM cum hazard curves for the association of global CBF with the secondary outcome in total (D), in the group with CAS<50% (E), and in the group with CAS ≥ 50% (F).
